# Author’s reply

**Published:** 2010

**Authors:** Shivam Sinha, S C Goel

**Affiliations:** *Department of Orthopedics, IPGMER, Kolkata, B-2, Brij Enclave, Sunderpur, Varanasi, India*; 1 *Department of Orthopedics, IMS BHU, Varanasi, A-23, Brij Enclave, Sunderpur, Varanasi, India*

Sir

We are grateful to Prof. Marcos Matos for showing interest in our study. We graded the gross examination and manual palpation based on the softness of callus formed. Histological examination of callus was done by longitudinally sectioning the bone including normal bone ends on both the sides.

Callus diameter was also measured by calipers and then compared with the contralateral unfractured normal ulna. Callus area could be obtained by subtracting the normal bone diameter from that of the fractured bone. Since this measurement was showing imprecision in between the observers, this parameter was not included in histomorphometric assessment, as what has been pointed out.^1^

The histological sections were decalcified and stained with Hematoxyline and Eosine as mentioned in materials and methods.

Thanks for bringing forward further details, which we missed to incorporate in the manuscript
